# Metal Artifact Reduction Around Cervical Spine Implant Using Diffusion Tensor Imaging at 3T: A Phantom Study

**DOI:** 10.21203/rs.3.rs-2665952/v1

**Published:** 2023-03-16

**Authors:** Slimane Tounekti, Mahdi Alizadeh, Devon Middleton, James S Harrop, Hiba Bassem, Laura Krisa, Choukri Mekkaoui, Feroze B. Mohamed

**Affiliations:** Thomas Jefferson University; Thomas Jefferson University; Thomas Jefferson University; Thomas Jefferson University; Institute for Cognitive Science; Thomas Jefferson University; Athinoula A. Martinos Center for Biomedical Imaging; Thomas Jefferson University

## Abstract

Diffusion MRI continues to play a key role in non-invasively assessing spinal cord integrity and pre-operative injury evaluation. However, post-operative Diffusion Tensor Imaging (DTI) acquisition of a patient with a metal implant results in severe geometric image distortion. A method has been proposed here to alleviate the technical challenges facing the acquisition of DTI in post-operative cases and to evaluate longitudinal therapeutics. The described technique is based on the combination of the reduced Field-Of-View (rFOV) strategy and the phase segmented acquisition scheme (rFOV-PS-EPI) for significantly mitigating metal-induced distortions.

A custom-built phantom based on spine model with metal implant was used to collect high-resolution DTI data at 3 Tesla scanner using a home-grown diffusion MRI pulse sequence, rFOV-PS-EPI, single-shot (rFOV-SS-EPI), and the conventional full FOV techniques including SS-EPI, PS-EPI, and the readout-segmented (RS-EPI).

This newly developed method provides high-resolution images with significant reduced metal-induced artifacts. In contrast to the other techniques, the rFOV-PS-EPI allows DTI measurement at the level of the metal hardware whereas the current rFOV-SS-EPI is useful when the metal is approximately 20 mm away. The developed approach enables high-resolution DTI in patients with metal implant.

## Introduction

Diffusion Tensor Imaging (DTI) has emerged as a key tool for in-vivo investigation of the central nervous system (i.e. brain and spinal cord (SC)) integrity and white matter (WM) connectivity mapping ^1,2^. It has the potential of probing changes in neural system microstructure with certain pathology by assessing the diffusion properties of water molecules inside the biological tissues in multiple directions, providing insights into the organization and orientation of structures ^3^. DTI-derived metrics, i.e., Fractional Anisotropy (FA), Mean Diffusivity (MD), Radial Diffusivity (RD), and Axial Diffusivity (AD), could be computed and used as a reliable imaging biomarker for describing the SC microstructure changes with certain pathology ^4^. In fact, numerous studies have demonstrated that there is a correlation between the computed metrics and the standard scales used for assessing the severity of physical disability such as the Japanese Orthopaedic Association (JOA) or the modified JOA (mJOA) score ^5,6^ as well the INSCSCI score.

Spinal Cord disorders such as spinal cord injury (SCI) and Degenerative Spondylotic Myelopathy (DSM) can affect the entire nervous system and lead to tissue/axonal damage, i.e., demyelination, transection, and atrophy, resulting in serious clinical complication including motor and/or sensory systems dysfunction, partial or complete paralysis, etc. In this context, the microstructural damages such as demyelination and axonal loss associated with SCI cause changes in the diffusivity of water in the spinal cord, and fiber bundles density, exhibiting changes in DTI-derived metrics according to the level and severity of the damage ^7–9^. Surgical intervention for SCI treatment entail implantation of metallic hardware (Anterior or Posterior) for maintaining SC stabilization and avoid further short-term or long-term complications ^10^.

Structural MRI techniques, i.e., T1- and T2-weighted imaging, and DTI have gained popularity in clinical practice compared to conventional radiology modalities (CT, X-Ray) for preoperative diagnosis and assess of SCI. Kara et al have demonstrated the feasibility of using DTI-derived indices as robust biomarkers for the early detection of DSM in patients with normal-appearing T2-weighted images ^11^. A recent study has shown that the change of DTI-metrics correlates with the change in the mJOA score as well as with the mJOA recovery rate, showing evidence that preoperative DTI has prognostic potential in predicting surgical outcomes ^12^. However, DTI is hampered by its intrinsic low-sensitivity, as well as the low-spatial resolution and high-sensitivity to motion, leading to image distortions and a weak signal-to-noise ratio (SNR). In addition, performing DTI on SC is technically challenging mainly due to the small dimensions of the SC, the physiological motion (e.g., heart, lungs, and throat in its proximity) and the susceptibility-induced distortions.

Even with these new improvements, postsurgical metallic implants typically induce dramatic magnetic field inhomogeneities, leading to severe image distortions. The metal-induced artifact depends on the implant hardware (material, size, shape), the magnetic field B0 strength, and MRI sequence ^13^. Moreover, DTI is often performed using the single-shot Echo Planar Imaging pulse sequence (SS-EPI) due to its fast acquisition speed. However, this acquisition method is prone to various limitations, including Eddy-current and high-sensitivity to magnetic field inhomogeneity, inducing artifacts dramatically affecting the image quality. Phase-segmented EPI (PS-EPI) and readout-segmented EPI (RS-EPI) pulse sequences have been introduced recently for performing high-resolution and distortion reduced DTI on the brain ^14–16^. Though, the application of these techniques for SC diffusion imaging remains limited due to their high sensitivity to motion.

Therefore, given these technical challenges limiting the potential of getting images near the metal hardware, the use of DTI for evaluating postoperative clinical outcomes remain an unexplored field and the post-surgery SCI assessment is still heavily based on structural MRI techniques and a surgeon’s skills. Studies have attempted various acquisition approaches to demonstrate the feasibility of performing metal-artifacts reduction for DTI scan SC near metal implant ^17–19^. Despite these recent technical developments, the potential to effectively suppress metal-induced artifacts for the diffusion-MRI scan is still unreached. This is mainly due to the limitations of the proposed techniques, including through-plane distortion, image blurring, and low SNR. In addition, this can be due to the high B0 field inhomogeneity in proximity to the metal, specifically at ultra-high field (UHF) such as 3 or 7 Tesla.

In this study, we propose a reduced-Field-Of-View phase-segmented EPI (rFOV-PS-EPI) diffusion weighted MR pulse sequence to address geometric distortions near the metal in DTI scan of SC at 3T. High-resolution distortion reduced diffusion-weighted images were collected on a custom-build cervical spine phantom model with a metal implant. The efficacy of the proposed pulse sequence in reduction of metal-artifacts was evaluated through the comparison with the rFOV-SS-EPI pulse sequence as well as the full-FOV approaches: SS-EPI, PS-EPI, and RS-EPI. To date, this is the first implementation of a rFOV-PS-EPI DTI sequence used to image spinal cord phantom model in the presence of metal-based spinal hardware currently used by surgeons.

## Material And Methods

### 1. Protocol Setup

The experiments in this study were conducted on 3T Magnetom Prisma system (Siemens Healthineers, Erlangen, Germany) with a gradient maximum amplitude of 80 mT/m and a maximum slew-rate of 200 T/m/s. The phantom was placed in the vendor 64-channel receive head coil, whereas the MR body coil was used for radiofrequency (RF) transmission. In this phantom-based study, no human or animal subjects were involved in phantom construction or data collected. Therefore, an approval from the ethics committee was not required.

### 2. Cervical Spine Phantom:

**Figure 1:**
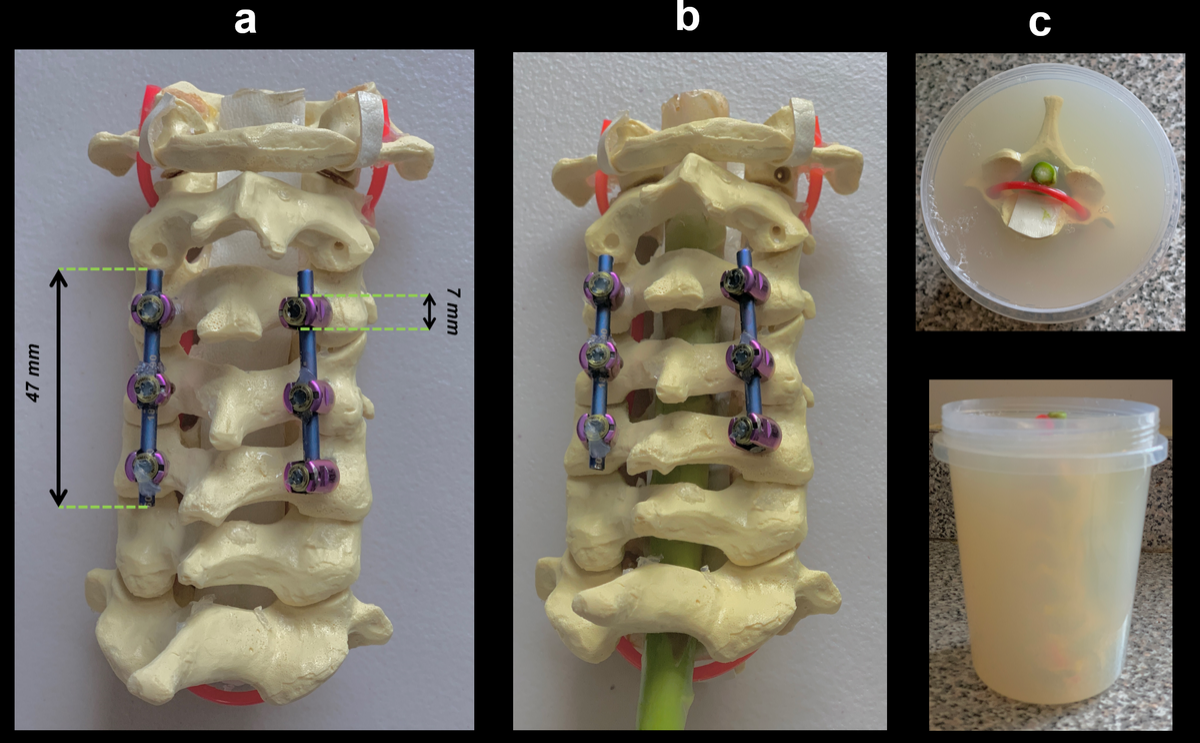
The cervical Spine phantom used in this study. (a) Spine model with MRI compatible Titanium Alloy implants. (b) An asperges was placed in the spinal canal of the spine model. (c) The spinal phantom model was centered in a cylindric plastic container and filled with the agar solution.

Figure. 1 illustrates the spine model as well as the final phantom used in the experiments. A cervical spine model with MRI compatible Titanium Alloy implants as currently used in the clinics was constructed and (NuVasive, CA, USA) was used in this study (Fig. 1. a). Ten gram of agar-agar powder was diluted in one liter of water to the phantom was suspended in this solution. An asparagus was used in the phantom to provide an anisotropic structure to contrast with the surrounding gel medium. It was placed in the spinal canal of the spine model (Fig. 1. b). The spinal phantom model was centered in a cylindric plastic container. After filling the container with the agar solution, it was left in ambient temperature to set (Fig. 1. c).

### 3. Pulse sequence design

**Figure 2:**
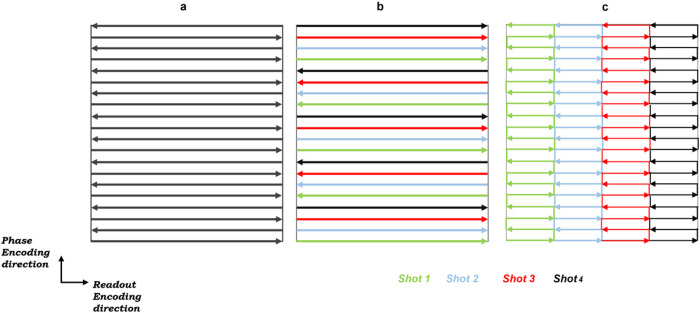
(a)The readout sampling scheme of the single-shot EPI (SS-EPI), (b) Phase-Segmented EPI (PS-EPI), and (c) the Readout-Segmented EPI (PS-EPI). The SS-EPI pulse sequence need one RF shot for data acquisition. However, the PS-EPI and RS-EPI (Figure 2. b and c) require multiple RF shots for fully sampling the Fourier-Space.

Figure. 2. illustrates the EPI readout sampling scheme of the SS-EPI, PS-EPI, and the RS-EPI. The gold standard SS-EPI pulse sequence allows collecting the data in one excitation pulse (Fig. 2. a). However, the PS-EPI (Fig. 2. b) and RS-EPI (Fig. 2. c) require multiple RF shots for fully sampling the Fourier-Space.

**Figure 3:**
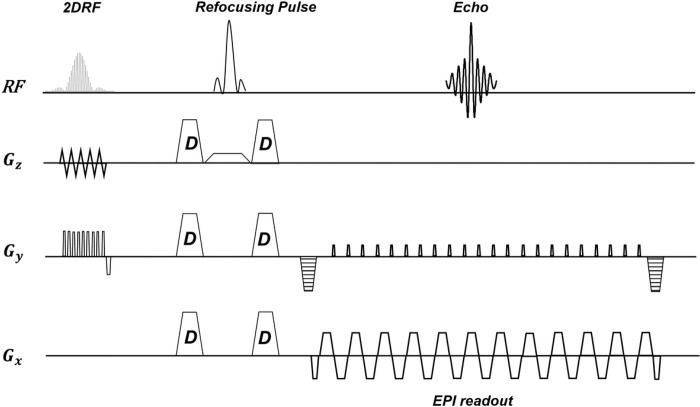
The diagram of the developed rFOV-PS-EPI pulse sequence used in this study. The 2DRF excitation pulse consists of 45 phase encoding blips with an echo spacing of 0.37 ms, resulting in a total duration of 16.93 ms.

In this study, the gold standard SS-EPI technique was modified to build the PS-EPI MR pulse sequence using the IDEA pulse sequence environment (Siemens Medical Solutions, Erlangen, Germany). Then, the 2DRF excitation pulse was implemented into the developed PS-EPI sequence to create the proposed rFOV-PS-EPI approach. The diagram of the rFOV-PS-EPI pulse sequence used for collecting data is displayed in Fig. 3. The 2DRF excitation pulse was used to acquire a rFOV along the Phase Encoding (PE) direction. It consists of 45 PE lines with an echo spacing of 0.37 ms, resulting in a total duration of 16.93 ms. The conventional Stejskal-Tanner diffusion preparation scheme with one refusing pulse of 180 degrees is used to acquire diffusion weighted images ^20^. Although the 2DRF aims to acquire a reduced number of acquired lines at each RF pulse, the Phase segmentation sampling scheme allows achieving a significantly short EPI readout train, addressing the susceptibility-induced geometric distortions. The fat saturation technique, not shown in the diagram, was applied prior the RF excitation pulse in order to suppress the unwanted fat signal and address the chemical-shift related artifacts in the images.

### 4. Acquisition Parameters

Twelve axial diffusion-weighted images were acquired with a b-value of 600 s/mm^2^ applied along twelve non-collinear diffusion encoding directions using the vendor direction file under MDDW diffusion mode. One non-DW image with b-value of 0 s/mm^2^ was collected prior the dMRI images. The detailed acquisition parameters of all diffusion MR pulse sequences used in this phantom study are given in Table. 1.

For assessing the effects of the EPI readout length on metal-induced artifacts, one axial image was collected with the rFOV-PS-EPI at b-value of 0 s/mm^2^ (b0-image) using the following parameters: TR = 2900 ms, TE = 60 ms, EPI factor = 3, number of segments = 19, bandwidth = 1286 Hz/Pixel, Slice thickness = 5 mm, FOV = 120×57 mm^2^, matrix size = 134×64 mm^2^, number of slices = 30, Fat sat region thickness = 20 mm, scan time = 1:07 minutes. In addition, a conventional anatomical 3D T2-weighted images were collected using a SPACE pulse sequence. The spatial resolution was set to 0.9×0.9×5 mm^3^. The other scan parameters are as fellow: TR = 1500 ms, TE = 110 ms, Flip angle = 120°, bandwidth = 620 Hz/Pixel, Slice thickness = 5 mm, FOV = 120×120 mm^2^, matrix size = 128×128 mm^2^, number of slices = 30, phase oversampling = 30%, slice Partial Fourier = 6/8, scan time = 1:23 minutes.

## Results

**Figure 4:**
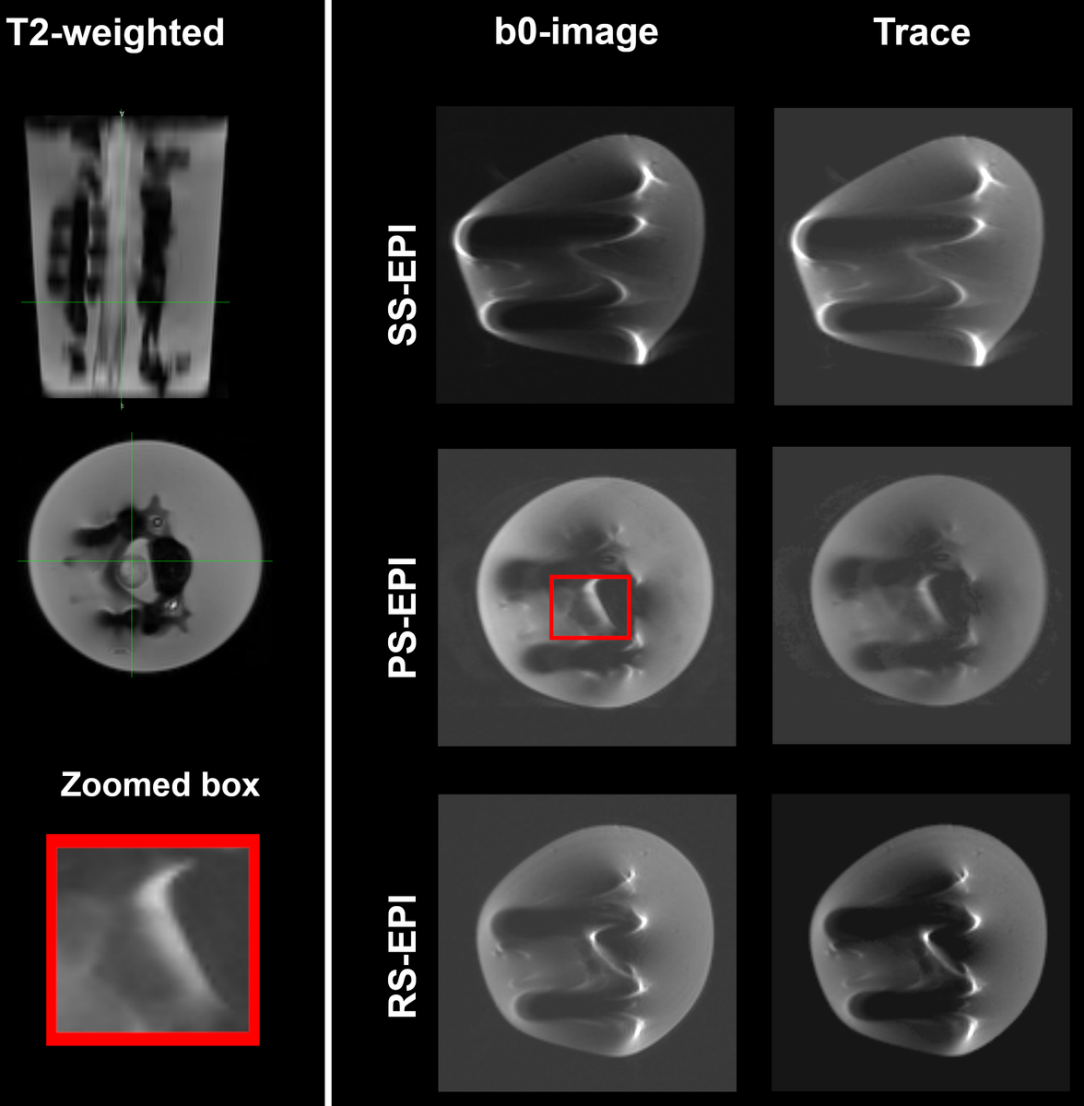
the b0-images and the Trace maps obtained using the standard SS-EPI, the PS-EPI, and the RS-EPI of the same slice. The selected slice is located at the metal hardware level as indicated by the green line in the sagittal view of the T2-weighted image. The SS-EPI sequence suffers from high-intensity susceptibility artifacts. The RS- and PS-EPI sequences significantly reduce the geometric distortions and maintain the spherical shape of the phantom container. However, the asparagus can be only detectable with the PS-EPI pulse sequence as highlighted in the zoomed red box.

Figure 4 displays the b0-images and the Trace maps obtained using the standard SS-EPI (top row), the PS-EPI (second row), and the RS-EPI (bottom row) of the same slice shown in the T2-image. The selected slice is located at the metal hardware level and indicated by the green line in the sagittal view of the T2-image. The gold standard SS-EPI sequence suffers from high-intensity susceptibility artifacts which affect the shape of the phantom and completely hinders the visibility of the asparagus. The RS- and PS-EPI sequences significantly reduce the geometric distortions due to the presence of metal, maintaining the spherical shape of the phantom. However, the asparagus-model can only be detectable and visible with the PS-EPI pulse sequence as highlighted in the zoomed red box.

**Figure 5:**
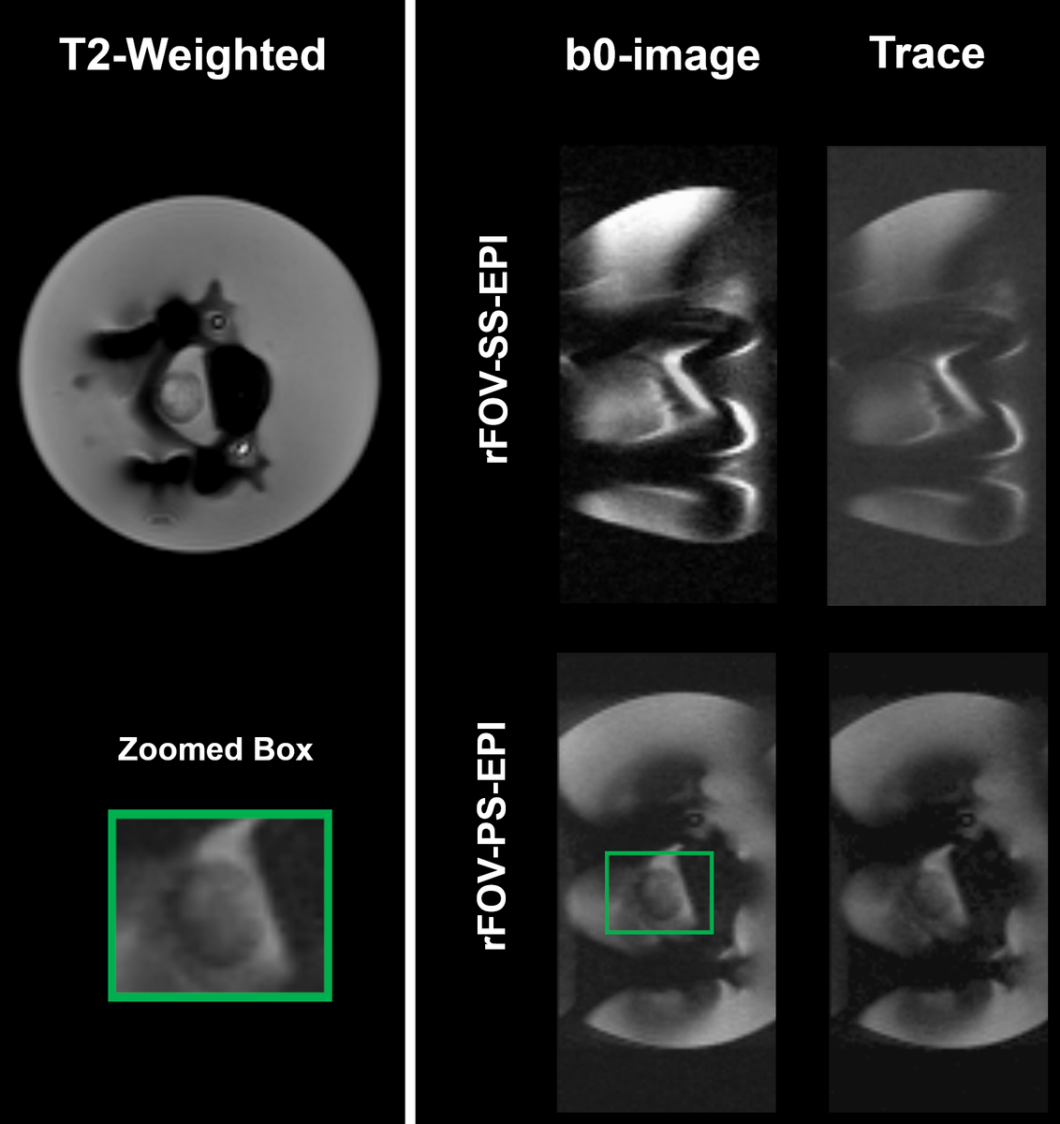
The b0-images and Trace maps computed from the rFOV-SS-EPI and the developed rFOV-PS-EPI pulse sequence of the slice at the metal level. The zoomed green box shows that the Spinal Canal as well as the asparagus spherical shape are clearly visible in the images obtained with the proposed rFOV-PS-EPI method.

Figure 5 shows the comparison between b0-images and Trace maps computed from the rFOV-SS-EPI, commonly used for SC imaging, and the developed rFOV-PS-EPI pulse sequence approximately at the same slice selected in Fig. 4. The Spinal Canal as well as the asparagus spherical shape are clearly visible in the images obtained with the proposed rFOV-PS-EPI method.

**Figure 6:**
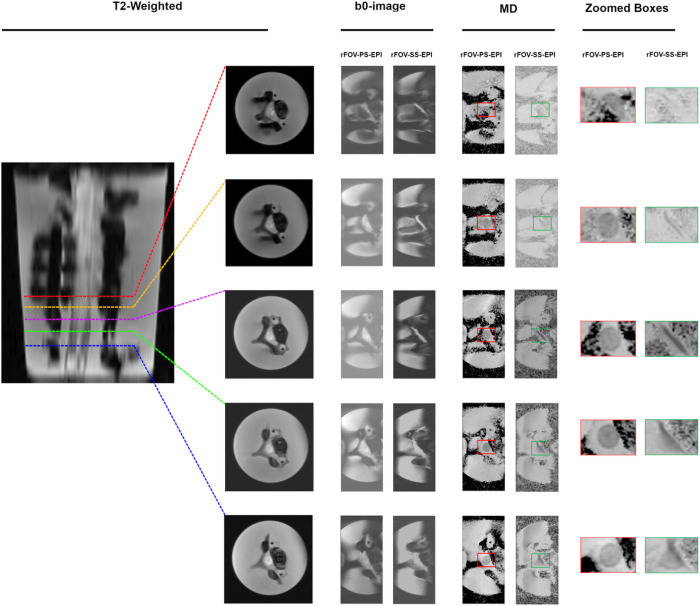
Axial views of the b0-images and MD maps obtained at 0 mm, 5 mm, 10, 15, 20 mm from the metal using the rFOV-PS-EPI and rFOV-SS-EPI pulse sequences. The selected slices are indicated by the dashed colored lines on the sagittal view of the T2-image and displayed on the right. The capacity of the developed method to obtain a true shape of the asparagus at b0 and MD maps at a slice level close to the metal implant is highlighted by the red zoomed boxes. The rFOV-SS-EPI provide a image of the asparagus at a distance of 20 mm away from the metal hardware.

In order to accurately assess the potential of suggested method in providing distortion-reduced images near the metal, Fig. 6 compares the b0-images and MD maps obtained at different distance from the metal (metal level, 5, 10, 15, 20 mm) using the rFOV-PS-EPI and rFOV-SS-EPI pulse sequences. The selected slices are indicated by the dashed colored lines on the sagittal view of the T2-image and displayed on the right. The ability of the developed method to obtain a true shape of the asparagus at b0 and MD maps at a slice level close to the metal implant is highlighted by the colored zoomed boxes.

**Figure 7:**
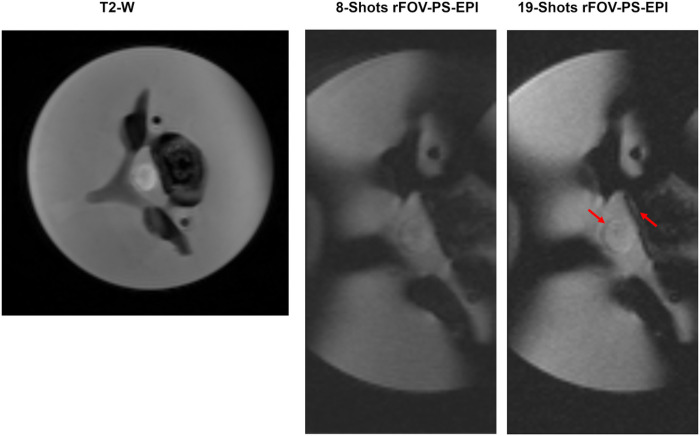
The effects of long echo train EPI readout on the image distortions. Two b0-images acquired with eight-shot and 19-shots. The visual comparison shows no difference in distortions level between the two images. However, the shorter echo train improves the SNR, allowing better detection of small structure such as the asparagus edges.

Figure 7 displays the effects of long echo train EPI readout on the image distortions. It illustrates two images acquired with an eight- and nineteen-EPI shots. The visual comparison shows no difference in distortions level between the two images. However, the shorter echo train improves the SNR, allowing better detection of small structure such as the asparagus edges as indicated by red arrows.

## Discussion

The utility of Diffusion Weighted Imaging (DWI) in pre-operative SCI has been demonstrated in several clinical studies. However, the limitations of DTI have been demonstrated in providing post-surgery information ^4^. High-intensity geometric distortions arise due to the presence of metal in the imaging plane or in the adjacent plane, also known respectively as in-plane and through-plane artifacts ^13,21^. It has been shown that the size and the intensity of metal-induced artifacts increase with magnetic field strength and employed pulse sequence parameters such as the echo spacing and the receiver bandwidth ^22^. Therefore, in this phantom-based study, a reduced FOV- based acquisition approach, termed rFOV-PS-EPI, is proposed to mitigate the metal-induced artifacts and to demonstrate the feasibility of performing DTI scans near metallic hardware at ultra-high field strength at 3T.

The b0-images and Trace maps obtained using the standard full FOV pulse sequence (SS-EPI) are dramatically distorted by the susceptibility artifacts (Fig. 4, first row), showing evidence of the vulnerability of this method to the metal-induced distortions and its inappropriacy for post-surgery SCI imaging. The segmented Fourier-space sampling scheme (in readout or phase direction) achieves shorter echo spacing and high bandwidth per pixel, reducing the severity of image distortions in the presence of metal (Fig. 4). The asparagus shape detected and zoomed in Fig. 4 illustrates that the PS-EPI pulse sequence addresses the image artifacts and significantly reduces the geometric distortions compared to SS-EPI and RS-EPI techniques.

In order to achieve a shorter echo-spacing and further improve the image quality, the reduced FOV technique was implemented into the PS-EPI method to create the proposed rFOV-PS-EPI pulse sequence. Although there is residual in-plane distortion and signal dropout at the level of the metal implant, the developed method shows great potential in obtaining reduced artifacts and high image quality of the asparagus model compared to the standard imaging technique rFOV-SS-EPI (Fig. 5).

The b0-images and MD maps show that using the rFOV-SS-EPI asparagus shape can be detected starting from the fifth slice away from the metal (~ 20 mm) (Fig. 6). However, it can be partially seen (~ 50%) near the spinal hardware and fully visible at the previous slice using the suggested rFOV-PS-EPI method as shown in the red boxes (Fig. 6). The efficacy of our technique is demonstrated by a significant reduction in through-plane image distortion at 5 mm slice thickness, commonly used to image spinal cord ^23^.

The use of high segmentation factor of 19-shot, resulting in an EPI factor of 3 does not provide an improvement in image distortions over the 8-shot sequence (EPI factor = 8, see Table 1) (Fig. 7). This might be due to the long Time to Echo (TE), i.e., 60 ms, imposed by the gradients’ performance and hardware limitations. Therefore, the use of a high-performance gradient system ^24^ may shorten TE in diffusion scans, further enhancing the image quality by reducing image artifacts and increasing SNR.

For improving the capacity of in-plane metal artifacts suppression, additional correction techniques such as View-Angle-Tilting (VAT) can be implemented into the proposed pulse sequence. However, it has been shown that VAT-EPI technique induces strong image blurring ^17^.

The suggested MR pulse sequence could provide distortion correction for high resolution DTI images near the titanium alloy, though some technical limitations should be highlighted. The major drawback of the proposed imaging strategy is the prolonged scan time associated with the multi-shot acquisition scheme ^25,26^. In this phantom experiment, the scan time of the 8-shot DTI scan was five times longer than the rFOV-SS-EPI. In addition, the cardiac gating technique is commonly used for *in vivo* DTI scan of the SC for reducing the contribution of the cardiovascular pulsations in signal corruption, resulting in increased total scan time.

Furthermore, high angular-resolution DTI as well as advanced diffusion models, such as Diffusion Kurtosis Imaging (DKI) and Neurite Orientation Density Dispersion Imaging (NODDI), ^9,27^ to fully characterize the SC microstructure enables thorough SC post-operative assessment, albeit an extended acquisition time.

Several studies have demonstrated that the multi-shot acquisition strategy is a reliable approach for high-resolution DTI, although it has high-sensitivity to motion-induced artifacts ^15, 28–31^. The Inter-shot phase variations can be amplified by the high-intensity diffusion gradients, resulting in severe ghosting artifacts. Different strategies, including navigator-based sequences ^32–35^, have also been considered to reduce physiologic noise.

## Conclusion

While the reduced FOV (rFOV-SS-EPI) acquisition technique has been conventionally used to perform high resolution DTI on SC, its efficacy for post-operative SCI evaluation remains limited by the presence of artifacts due to metal implants. Here, we introduce a technique combining the reduced FOV strategy and multi-shot acquisition scheme. The phantom-based results demonstrate the benefit of the proposed acquisition method in achieving high-resolution DTI with reduced geometric distortion near the metal-based spinal hardware at 3T. Future work will involve validation of this sequence in post-operative patients with metal implants.

## Figures and Tables

**Table.1:** DTI pulse sequence parameters used on Phantom

Parameters	SS-EPI	PS-EPI	RS-EPI	rFOV-SS-EPI	rFOV-PS-EPI
TR (ms)	5200	2600	5370	4500	2900
TE (ms)	90	43	75	62	60
Number of slices	30	30	30	30	30
Slice thickness (mm)	5	5	5	5	5
FOV (mm^2^)	120×120	120×120	120×120	120×57	120×57
Matrix size (mm^2^)	134×134	134×134	134×134	134×64	134×64
Spatial resolution (mm^3^)	0.9×0.9×5	0.9×0.9×5	0.9×0.9×5	0.9×0.9×5	0.9×0.9×5
PE direction	R > L	R > L	R > L	R > L	R > L
Phase Partial Fourier	6/8	6/8	×	6/8	6/8
Read Partial Fourier	×	×	6/8	×	×
Echo spacing (ms)	1	1.11	0.38	0.98	1.1
Bandwidth (Hz/Px)	1244	1382	691	1166	1286
EPI factor	134	19	134	64	8
Number of segments	1	7	7	1	8
Fat region Thickness (mm)	×	×	×	20	20
Scan Time (min)	1:13	4:36	6	1:03	5:07

## Data Availability

Collected T2, Diffusion MRI images, computed maps such as FA, MD will be made available by Slimane Tounekti upon request.
